# Supplementary material for: Ultra-fast vortex motion in a direct-write Nb-C superconductor
Source: Nat Commun. 2020 Jul 3;11:3291. doi: 10.1038/s41467-020-16987-y (PMC7335109; doi:10.1038/s41467-020-16987-y)
Supplement: Supplementary file 1 — Supplementary Information [file 41467_2020_16987_MOESM1_ESM.pdf]

Supplementary information

Ultra-fast vortex motion in a direct-write Nb-C superconductor

O. V. Dobrovolskiy et al.

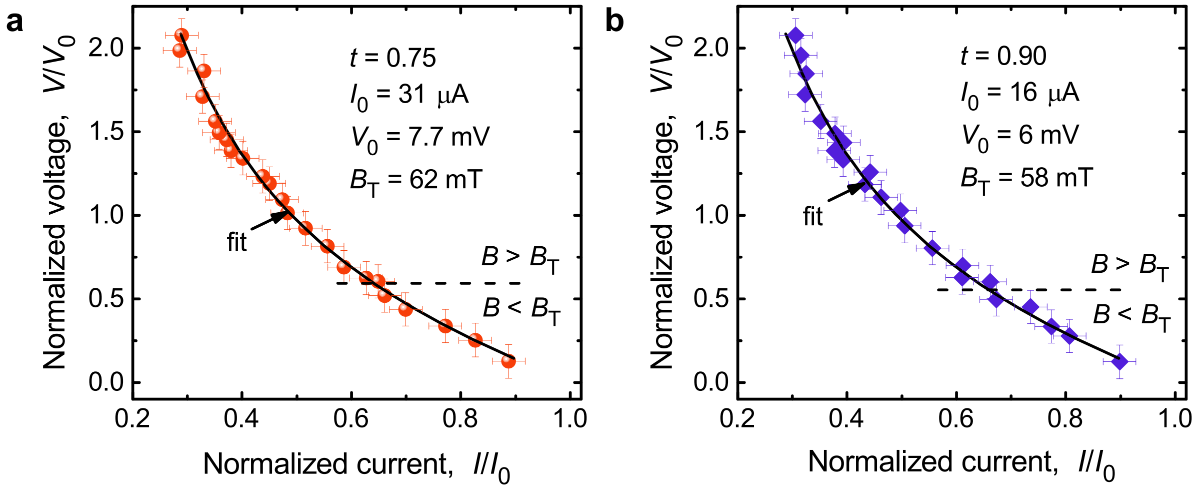

Supplementary Figure 1: Complete set of instability points. **a** $T = 0.75T_c$. **b** $T = 0.9T_c$. Symbols: experiment; solid lines: fits to Supplementary Eq. (1) with the fitting parameters as indicated. The corresponding error bars are the standard error of the mean. Source data are provided as a Source Data file.

Supplementary Note 1

For $10\,\mathrm{mT} \lesssim B \lesssim 100\,\mathrm{mT}$, the dependence of the critical current is described well by the dependence $I_c(B) = I_c(0\,\mathrm{T})B_{\mathrm{stop}}/2B$ and $I_c(B)$ exhibits a linear decrease at low fields. Though the dependence $I_c \propto 1/(B_0 + B)$ of the Kim model of bulk pinning [1] could, in principle, describe a linear dependence $I_c(B)$ at fields below some field B_0 and $I_c \propto 1/B$ at $B \gg B_0$, the totality of our experimental data indicates the dominating role of the edge mechanism [2] of vortex pinning in the studied sample at $B \lesssim 100\,\mathrm{mT}$. We note that for our microstrip a current $I_c \simeq 10\,\mu\mathrm{A}$ induces a self-field $B_{\mathrm{self}} = 0.5\mu_0 I w^{-1} \ln(2w/d) \simeq 10^{-2}\,\mathrm{mT}$ which is much smaller than $B_{\mathrm{stop}} \simeq 10\,\mathrm{mT}$ and, hence, the contribution of possible self-field effects to the observed crossover in $I_c(B)$ at $B \approx 10\,\mathrm{mT}$ is negligibly small. At larger fields, $B \gtrsim 100\,\mathrm{mT}$, a further crossover at B^* to a slower decrease of $I_c(B)$ as $B^{-0.5}$ is observed. This dependence, which follows from many models of volume pinning, can be explained by the increasing role of the volume pinning at higher vortex densities at larger magnetic fields. The assumed origin of the intrinsic pinning is the order parameter suppression at the grain boundaries of individual crystallites in the Nb-C-FIBID microstrip. In the studied microstrip I_c is close to I_{dep} and this is why one can conclude that intrinsic defects do not influence I_c too much. At the same time, the absence of a smoothing of $I_c(B)$ at $B \ll B_{\mathrm{stop}}$

suggests that a certain amount of edge defects can still be present in the microstrip. In general, this smoothing is expected for samples with ideal edge barriers due to the pair-breaking effect of the depairing current [3,4]. Accordingly, edge defects may influence not only I_c but also I^* of the microstrip. Theoretically, this effect was studied for a photon-induced hot spot playing the role of a defect [5]. It was revealed that the defect practically does not influence I^* if the defect is far from the edge where vortices enter the strip [5]. If the defect is located close to the edge where vortices enter the strip, a weak suppression of I^* is expected at low B when the size of the defect is smaller or comparable with the width of the vortex-free region in the microstrip [5]. Such a behavior is a direct consequence of the edge-controlled FFI [5]. Finally, we would like to note that, in principle, jumps on the I-V curves could also result from the hot spot formation unrelated to the FFI. However, in the hot spot model the dissipated power at the instability point I^*V^* does not depend on B whereas in our experiment it does, as is peculiar to the FFI [6–8]. Accordingly, the obtained experimental data cannot be explained by the hot spot model alone and both Joule heating and the LO-like FFI mechanism need to be taken into account.

Supplementary Discussion

We compared the deduced instability parameters with the Bezuglyj-Shklovskij (BS) theoretical model [9]. In the BS work [9], a scaling law was introduced for the electric field strength E^* and the current density j^* at the instability point

$$\frac{E^*}{E_0} = (1 - t(b)) \left(\frac{j^*}{j_0}\right)^{-1}. \tag{1}$$

In Supplementary Eq. (1), parameters E_0 and j_0 are defined as

$$\begin{aligned} E_0 &= 1.02 B_\mathrm{T} (D/\tau_\epsilon)^{1/2} (1 - T/T_\mathrm{c})^{1/4}, \\ j_0 &= 2.62 (\sigma_\mathrm{n}/e)(D\tau_\epsilon)^{-1/2} k_\mathrm{B} T_\mathrm{c} (1 - T/T_\mathrm{c})^{3/4}, \end{aligned} \tag{2}$$

$t = [1 + b + (b^2 + 8b + 4)^{1/2}]/3(1 + 2b)$ and $b = B/B_\mathrm{T}$ is the magnetic field normalized by the parameter

$$B_\mathrm{T} = 0.374 k_\mathrm{B}^{-1} e R_\Box h \tau_\epsilon. \tag{3}$$

In Supplementary Eq. (3), h is the heat removal coefficient and τ_ϵ is the quasiparticle energy relaxation time. The parameter B_T separates the region of small fields $B \lesssim B_\mathrm{T}$ at which heat

removal is fast enough and the instability is of non-thermal nature from the region of large fields $B_\mathrm{T} \lesssim B \lesssim 0.4 B_\mathrm{c2}$ with insufficient heat removal and the heating mechanism dominating the instability.

The curves calculated by Supplementary Eqs. (1) and (2) are shown in Supplementary Figure 1 by solid lines. The theoretical curves nicely fit the experimentally measured instability points in the normalized voltage $V^*/V_0 = E^*/E_0$ versus normalized current $I^*/I_0 = j^*/j_0$ representation with the fitting parameters $B_\mathrm{T} = 62\,\mathrm{mT}$, $V_0 = 7.7\,\mathrm{mV}$ and $I_0 = 31\,\mu\mathrm{A}$ at $0.75T_\mathrm{c}$, and $B_\mathrm{T} = 58\,\mathrm{mT}$, $V_0 = 6.0\,\mathrm{mV}$ and $I_0 = 16\,\mu\mathrm{A}$ at $0.9T_\mathrm{c}$. Here, the field- and temperature-dependent instability currents I^* and voltages V^* are determined from the I-V curves. From the specific power at the instability point, $P_0 = j_0 E_0 = (h/d)(T_\mathrm{c} - T)$ [9–12], following from Supplementary Eqs. (1)–(3) with $\sigma_n = 1/(R_\square d)$, one can deduce the heat removal coefficient $h \approx 2.6\,\mathrm{WK^{-1}cm^{-2}}$. Substitution of h and B_T into Supplementary Eq. (3) yields the energy relaxation time $\tau_\epsilon \approx 1.4\,\mathrm{ps}$. if one associates τ_ϵ with the electron-phonon scattering time τ_ep in the Larkin-Ovchinnikov model, the deduced τ_ϵ is at least one order of magnitude smaller than one could expect from τ_ϵ found in similar low-T_c highly disordered superconductors [13–15].

The deduced heat removal coefficient $h \approx 2.6\,\mathrm{WK^{-1}cm^{-2}}$ is of the same order of magnitude as for dirty Nb films on sapphire substrates [11], and it is one to two orders of magnitude smaller than h values for epitaxial BSSCO films on $\mathrm{SrTiO_3}$ substrates [16] and epitaxial YBaCuO films on sapphire substrates [17]. We assume that the presence of the Nb-C-FEBID layer on top of Nb-C-FIBID may have improved the effective heat removal from the sample.

Supplementary References

1. Kim, Y. B. & Stephen, M. J. *Superconductivity vol. 2*, 1107 (Marcel Dekker New York, 1969).

2. Plourde, B. L. T. *et al.* Influence of edge barriers on vortex dynamics in thin weak-pinning superconducting strips. *Phys. Rev. B* **64**, 014503 (2001).

3. Andratskii, V. P., Grundel, L. M., Gubankov, V. N. & Pavlov, N. B. Destruction of superconductivity in thin narrow films by a current. *Sov. Phys. JETP* **38**, 797 (1974).

4. Maksimova, G. M., Zhelezina, N. V. & Maksimov, I. L. Critical current and negative magnetoresistance of superconducting film with edge barrier. *Europhys. Lett.* **53**, 639–645 (2001).

5. Vodolazov, D. Y. & Klapwijk, T. M. Photon-triggered instability in the flux flow regime of a strongly disordered superconducting strip. *Phys. Rev. B* **100**, 064507 (2019).

6. Silhanek, A. V. *et al.* Influence of artificial pinning on vortex lattice instability in superconducting films. *New J. Phys.* **14**, 053006 (2012).

7. Grimaldi, G. *et al.* Speed limit to the Abrikosov lattice in mesoscopic superconductors. *Phys. Rev. B* **92**, 024513 (2015).

8. Shklovskij, V. A., Nazipova, A. P. & Dobrovolskiy, O. V. Pinning effects on self-heating and flux-flow instability in superconducting films near T_c. *Phys. Rev. B* **95**, 184517 (2017).

9. Bezuglyj, A. & Shklovskij, V. Effect of self-heating on flux flow instability in a superconductor near T_c. *Physica C* **202**, 234 (1992).

10. Lefloch, F., Hoffmann, C. & Demolliens, O. Nonlinear flux flow in TiN superconducting thin film. *Physica C* **319**, 258–266 (1999).

11. Peroz, C. & Villard, C. Flux flow properties of niobium thin films in clean and dirty superconducting limits. *Phys. Rev. B* **72**, 014515–1–6 (2005).

12. Bezuglyj, A. I. *et al.* Local flux-flow instability in superconducting films near T_c. *Phys. Rev. B* **99**, 174518 (2019).

13. Babic, D., Bentner, J., Sürgers, C. & Strunk, C. Flux-flow instabilities in amorphous $Nb_{0.7}Ge_{0.3}$ microbridges. *Phys. Rev. B* **69**, 092510–1–4 (2004).

14. Sidorova, M. V. *et al.* Nonbolometric bottleneck in electron-phonon relaxation in ultrathin WSi films. *Phys. Rev. B* **97**, 184512 (2018).

15. Sidorova, M. *et al.* Electron energy relaxation in disordered superconducting NbN films. *arXiv:1907.05039* (2019).

16. Xiao, Z. L., Voss-de Haan, P., Jakob, G. & Adrian, H. Voltage jumps in current-voltage characteristics of $Bi_2Sr_2CaCu_2O_{8+\delta}$ superconducting films: Evidence for flux-flow instability under the influence of self-heating. *Phys. Rev. B* **57**, R736–R739 (1998).

17. Xiao, Z. L., Andrei, E. Y. & Ziemann, P. Coexistence of the hot-spot effect and flux-flow instability in high-T_c superconducting films. *Phys. Rev. B* **58**, 11185–11188 (1998).
